# Longer time to antibiotics and higher mortality among septic patients with non-specific presentations -a cross sectional study of Emergency Department patients indicating that a screening tool may improve identification

**DOI:** 10.1186/s13049-015-0193-0

**Published:** 2016-01-06

**Authors:** Ulrika Margareta Wallgren, Viktor Erik Antonsson, Maaret Kaarina Castrén, Lisa Kurland

**Affiliations:** Karolinska Institutet, Department of Clinical Science and Education, Södersjukhuset, Sjukhusbacken 10, SE 118 83 Stockholm, Sweden; Fisksätra Vårdcentral (Primary Health Care Center), Fisksätra torg 20, SE 133 41 Saltsjöbaden, Sweden; Department of Emergency Medicine and Services, Helsinki University Hospital and Helsinki University, Haartmaninkatu 4, PL 340, 00029 HUS Helsinki, Finland; Section of Emergency Medicine, Södersjukhuset, Sjukhusbacken 10, SE 118 83 Stockholm, Sweden

**Keywords:** Sepsis, Emergency Department, Decreased General Condition

## Abstract

**Background:**

The presentation of sepsis is varied and our hypotheses were that septic patients with non-specific presentations such as decreased general condition (DGC) have a less favourable outcome, and that a screening tool could increase identification of these patients. We aimed to: 1) assess time to antibiotics and in-hospital mortality among septic patients with ED chief complaint DGC, as compared with septic patients with other ED chief complaints, and 2) determine whether a screening tool could improve identification of septic patients with non-specific presentations such as DGC.

**Methods:**

Cross sectional study comparing time to antibiotics (Mann Whitney and Kaplan-Meier tests), and in-hospital mortality (logistic regression), between 61 septic patients with ED chief complaint DGC and 516 septic patients with other ED chief complaints. The sensitivity and specificity of the modified Robson screening tool was compared with that of ED doctor clinical judgment (McNemar’s two related samples test) among 122 patients presenting to the ED with chief complaint DGC, of which 61 were discharged with ICD code sepsis.

**Results:**

Septic patients presenting to the ED with the chief complaint DGC had a longer median time to antibiotics (05:26 h:minutes; IQR 4:00–10:40, vs. 03:56 h:minutes; IQR 2:21–7:32) and an increased in-hospital mortality (crude OR = 4.01; 95 % CI, 2.19–7.32), compared to septic patients with other ED chief complaints. This association remained significant when adjusting for sex, age, priority, comorbidity and fulfilment of the Robson score (OR 4.31; 95 % CI, 2.12–8.77). The modified Robson screening tool had a higher sensitivity (63.0 vs. 24.6 %, *p* < 0.001), but a lower specificity (68.3 vs. 100.0 %, *p* < 0.001), as compared to clinical judgment.

**Discussion:**

This is, to the best of our knowledge, the first study comparing outcome of septic patients according to ED chief complaint. Septic patients presenting with a non-specific ED presentation, here exemplified as the chief complaint DGC, have a less favourable outcome. Our results indicate that implementation of a screening tool may increase the identification of septic patients.

**Conclusions:**

The results indicate that septic patients presenting with ED chief complaint DGC constitute a vulnerable patient group with delayed time to antibiotics and high in-hospital mortality. Furthermore, the results support that implementation of a screening tool may be beneficial to improve identification of these patients.

## Background

Sepsis is common [1, 2], and associated with high mortality [3–6]. The 19-32 % mortality of severe sepsis and septic shock [3–6] exceeds that of 8 % among patients suffering a myocardial infarction [7]. Time to antibiotic treatment is crucial for the outcome of septic patients [8, 9]. However, the clinical presentation of sepsis is often non-specific [10, 11], which may complicate identification [12] and lead to a delayed treatment and hence worsened prognosis.

Emergency Department (ED) identification of sepsis is based on clinical judgment, in turn, based on experience and diagnostic criteria according to guidelines [13, 14]. However, the sensitivity of clinical judgment has previously been shown to be low [15–17] and septic patients are not identified [15, 16]. Conversely, the sensitivity of a screening tool [18] has previously been shown to be superior to clinical judgment with respect to sepsis identification in the pre-hospital setting [17].

The primary aim of the current study was to assess the time to antibiotics and the in-hospital mortality rate among septic patients with non-specific ED presentations, as compared with septic patients with other presentations. Chief complaint Decreased General Condition (DGC) upon ED arrival was chosen as an example of a non-specific ED presentation. The second aim was to determine whether a screening tool [18] would increase the identification of sepsis among patients presenting to the ED with chief complaint DGC. The Robson screening tool [17, 18], originally created for pre-hospital use, is based on bedside diagnostics which makes it feasible for use in both pre-hospital and ED settings.

## Methods

### Study design

This is a retrospective cross sectional study of three groups of adult patients presenting to the ED at Södersjukhuset, Sweden. These groups are; septic patients presenting with the chief complaint DGC, septic patients with other ED chief complaints, and non-septic patients presenting to the ED with chief complaint DGC. The chief complaint is documented by the triage nurse in the ED.

ICD-10-code (International Classification of Diseases, Tenth Revision) sepsis upon discharge was used as reference for sepsis, and the codes used to include patients with sepsis were: A02.1, A22.7, A26.7, A32.7, A39.2, A39.4, A40.0–40.3, A40.8–41.5, A41.8–41.9, B37.7, R57.2, R65.0–65.1 [17]. Coding was generally performed by doctors when writing the summary for the period of hospital care upon discharge. Specially trained secretaries perform the actual registration of set diagnoses in the IT system and sometimes make supplementary adjustments, eg adding codes for interventions.

We compared time to antibiotics and in-hospital mortality between septic patients presenting with ED chief complaint DGC and septic patients with other ED chief complaints. Furthermore, the sensitivity and specificity of the Robson screening tool was compared with that of the ED doctor clinical judgment (as measured by chart review), with respect to sepsis identification, among patients presenting to the ED with chief complaint DGC.

### Study setting and population

The patients in the study were admitted to Södersjukhuset which is an urban, 676-bed teaching hospital with more than 120,000 adult Emergency Department (ED) visits annually. The ED is staffed by a few specialized emergency physicians, residents employed by the ED and residents and specialists from other specialties. Three groups of adult patients (≥18 years of age), admitted to the ED during the period of January 15th and December 31st 2008, were included.

**Fig. 1:**
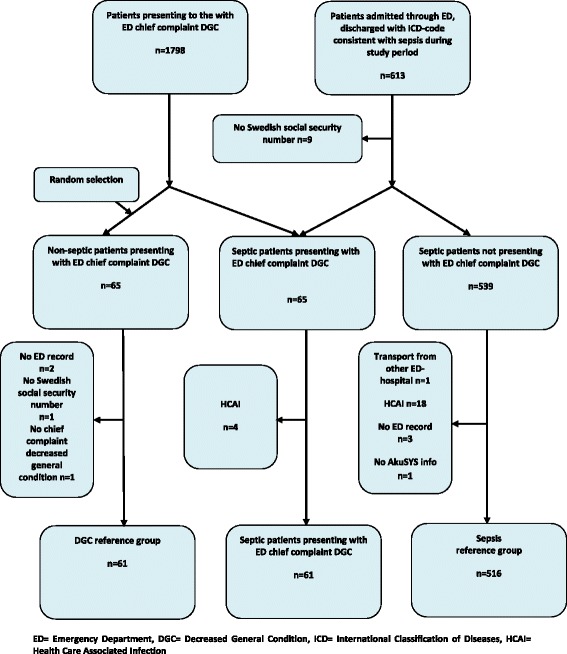
Flow chart for inclusion and exclusion. ED = Emergency Department, DGC = Decreased General Condition, ICD = International Classification of Diseases, HCAI = Health Care Associated Infection

For inclusion and exclusion of patients into the three groups, see Fig. 1. First, we identified all patients, 18 years or older, discharged from in-hospital care with an ICD-10-code compatible with sepsis, according to the in-hospital record system (Pasett, Sweden, Version 1.61). The septic group was further divided into two subgroups. Patients presenting to the ED with chief complaint DGC, according to the triage nurse and predefined triage categories documented in the ED electronical ledger AkuSys (AkuSys, Sweden, Version 5.5b), are referred to as septic patients presenting with ED chief complaint DGC. Septic patients presenting to the ED with chief complaints other than DGC, are referred to as the sepsis reference group.

Second, all patients 18 years or older, presenting to the ED during the study period with DGC as a chief complaint (according to AkuSys), were identified. A sample without sepsis as a discharge diagnosis was randomly selected by the SPSS program (Statistical Package for the Social Sciences, Inc., Chicago IL, version 21, 2012). This group is referred to as the DGC reference group, and the size was selected to be equal to the group of septic patients presenting with ED chief complaint DGC.

Only patients presenting with symptoms compatible with ongoing infection according to manual ED chart review were included in the two groups with outcome sepsis. Patients with Healthcare-Associated Infections (HCAI), defined as onset of infection ≥48 h after ED admission [19], were excluded from these two groups. In all groups, only patients admitted to in-hospital care via the ED were included, and exclusion criteria in all groups were: lack of ED admission record/ Swedish social security number/ AkuSYS data, and transport from other Emergency hospital of a patient already under treatment for sepsis (see Fig. 1 for inclusion and exclusion). Repeated ED visits during the study period for the same patient were included.

### Study procedure

#### Time to antibiotics

Time to antibiotics was defined as the time from ED arrival to the time of antibiotic administration, according to documentation in the patient chart. If antibiotic treatment had been initiated prior to ED admission, and no changes were done regarding choice of antibiotic type, the patient was excluded from the analysis of time to antibiotics.

#### Antibiotics

No specific guidelines are available regarding recommended types of antibiotics among septic patients without severe sepsis/ septic shock. We chose to include administration of both oral and intravenous antibiotics since Swedish guidelines [20] include oral antibiotics in the treatment of, for example, pneumonia known to be one of the most frequent causes of sepsis [12].

#### Mortality

In-hospital mortality was defined on the basis of hospital discharge code according to the in- the in-hospital record system Pasett.

#### The Robson screening tool

In accordance with the Robson screening tool [17, 18], a patient is considered septic if any two of the following criteria are present and new to the patient: temperature >38.3 °C or <36.0 °C, heart rate >90/min, respiratory rate >20/min, acutely altered mental status, glucose >6.6 mmol/l (unless diabetic) and the history is suggestive of a new infection.

The definition of an “acutely altered mental status” was not specified in the original Robson publication [18]. We considered this criterion to be fulfilled in accordance to the previous study by Wallgren et al. [17].

A “history suggestive of a new infection” was defined in accordance to the definition described in the previous pre-hospital study by Wallgren et al. [17] with the following additions; ED doctor ordering blood cultures or ED doctor ordering antibiotics.

We recorded the first documented ED measure of respiratory rate, temperature, heart frequency and glucose. Plasma glucose was used in the current study since this was standard in Sweden. According to the original Robson publication [18], we restricted the criterion plasma glucose above 6.6 mmol/l to non-diabetics. Diabetes was defined as a documented history of diabetes in the ED admission record.

We did not follow the second part of the original Robson tool [18], designed to screen for severe sepsis, since our aim was to study all septic patients (not only those with organ failure).

#### Clinical judgment of sepsis by ED doctor

Clinical judgment of sepsis by the ED doctor was defined as documentation of any of the following; “sepsis”/“septic”/“suspected sepsis”/“septicaemia”/”urosepsis”/”septic shock” or “septic syndrome” in the ED admission records.

### Data collection and handling

Data related to ED arrival, age, triage priority, gender and chief complaint were retrieved from the electronic ED ledger Akusys.

Information regarding ED doctor clinical judgment and pre-existing comorbidity was acquired from the ED admission records (Melior, Version 1.5, Siemens AB), which were also screened for signs consistent with infection [17].

Vital signs and time of initiation of antibiotics were obtained primarily from the, by a nurse handwritten and scanned, ED arrival chart (KoVis, Version 5.0, Global 360, Inc, via Melior). If missing there, vital signs were obtained from the, by a physician documented ED record and time of antibiotics from the list of medications for the care episode, reached through KoVis or Melior.

### Statistics

Median and interquartile range (IQR) were used to describe age and vital parameters, since these values were not normally distributed. To identify differences in patient characteristics between septic patients presenting with ED chief complaint DGC and the DGC reference group, as well as between septic patients presenting with ED chief complaint DGC and the sepsis reference group respectively, we used Fischer’s exact test for categorical variables (gender, triage priority, history of infection, fulfilment of Robson, clinical judgment sepsis, mortality) and a Mann–Whitney U test for numeric variables (age, vital parameters, plasma glucose). To compare the sensitivity and specificity of clinical judgment with that of Robson sepsis screening tool we used McNemar’s test. Time to antibiotics was compared between septic patients presenting with ED chief complaint DGC and the sepsis reference group by using a Mann–Whitney U test and a Kaplan Meier test. Mortality rates between the same groups were compared by logistic regression adjusting for sex, age, priority, Charlson comorbidity score [21] and fulfilment of Robson [18]. The final regression model was tested using the Hosmer-Lemeshow goodness-of-fit test. Data was analyzed using SPSS (Version 22, IBM Company, Chicago, IL, USA) statistical software. A *p* value of <0.05 was considered statistically significant.

### Ethical approval

Stockholm Regional Ethical Review Board approval was obtained for this study and a waiver of informed consent was granted.

## Results

During the study period, 1798 ED visits presenting with ED chief complaint DGC were admitted to in-hospital care. Of these, 65 (3.6 %) were discharged from in-hospital care with an ICD-code consistent with sepsis. Four of these were excluded due to HCAI, leaving a total of 61 patients in the group of septic patients presenting with ED chief complaint DGC.

Of the total of 613 patients admitted through the ED and discharged with sepsis, nine lacked a Swedish social security number, 65 presented with ED chief complaint DGC and twenty-three were further excluded for various reasons (see Fig. 1), leaving a total of 516 patients in the sepsis reference group. For characteristics of the first two groups, see Table 1 and Table 2.

**Table 1:** Characteristics of the 61 septic patients presenting to the ED with chief complaint DGC and the 61 patients in the DGC reference group

	*Septic patients presenting with ED chief complaint DGC*		*Sepsis reference group*		
	*n* = 61		*n* = 516		
Variable	Median (IQR)	Number (%^a^)	Median (IQR)	Number (%^a^)	P-value
Age, yr	78(67**–**85)	61(100.0)	73(61**–**82)	516(100.0)	.013
Gender					.224
-male		39(63.9)		299 (57.9)	
ED Vital parameters					
-Respiratory rate breaths/min)	24 (20**–**26)		22(18**–**28)		.751
-Oxygen saturation (%)oxygen	94 (90**–**96)		94(91**–**97)		.365
-Heart rate (beats/min)	89(78**–**107)		95(80**–**113)		.229
-Temperature (°C)	37.3(36.7**–**38.1)		38.2(37.3**–**39.1)		.001
-Systolic BP	120(107**–**145)		132(110**–**155)		.030
-Altered mental status		31 (50.8)		143(27.7)	.000
ED Plasma Glucose, mmol/l	6.9(6.1**–**7.8)		7.3(6.2**–**9.4)		.076
History of infection		55(90.2)		505(97.9)	.005
Fulfilment of Robson^b^		34(63.0)		286(73.5)	.074
Clinical judgment sepsis		15(24.6)		166(32.2)	.144
Diabetes		9(14.8)		96(18.6)	.294
History of alcohol overconsumption		17(27.9)		40(7.8)	.000
Antibiotics prior to admittance		2(3.3)		91(17.6)	.003
ED Triage priority					.069
-Red (%)		5(8.2)		81(15.7)	
-Orange (%)		18(29.5)		210(40.7)	
-Yellow (%)		32(52.5)		185(35.9)	
-Green (%)		6(9.8)		39(7.6)	
-Blue (%)		0(0.0)		1(0.2)	
Charlson comorbidity score	2.0(0.0**–**3.0)		2.0(0.5**–**3.0)		.848
In-hospital mortality		20(32.8)		56(10.9)	.000

*DGC* decreased general condition; *ED* emergency department; *IQR* interquartil range; *BP* blood pressure

^a^of patients with documentation of parameter

^b^A history suggestive of a new infection and fulfilment of two or more of the following criteria: temperature < 36°C or > 38.3°C, heart rate >90 beats/min, respiratory rate >20 breaths/min, acutely altered mental status, plasma glucose >6.6 mmol/L (unless diabetic) is considered positive for sepsis. All necessary parameters are documented

**Table 2:** Characteristics of 61 septic patients presenting to the ED with chief complaint DGC and 516 septic patients with other ED presentations (the sepsis reference group)

	*Septic patients presenting with ED chief complaint DGC*		*DGC reference group*		
	*n* = 61		*n* = 61		
Variable	Median (IQR)	Number (%^a^)	Median (IQR)	Number (%^a^)	*P*-value
Age, year	78(67–85)		80(71–88)		.140
Gender					.034
-male		39(63.9)		28(45.9)	
ED Vital parameters					
-Respiratory rate (breaths/min)	24 (20–26)		20(16–24)		.001
-Oxygen saturation (%)oxygen	94 (90–96)		96(93–98)		.005
-Heart rate (beats/min)	89(78–107)		81(68–89)		.002
-Temperature (°C)	37.3(36.7–38.1)		36.9(36.3–37.2)		.001
-Systolic blood pressure	120(107–145)		135(120–160)		.007
-Altered mental status		31 (50.8)		15(24.6)	.005
ED Plasma Glucose mmol/l	6.9 (6.1–7.8)		6.0(5.2–7.7)		.015
History of infection		55(90.2)		33(54.1)	.000
Fulfilment of Robson^b^		34(63.0)		13(31.7)	.002
Clinical judgment sepsis		15(24.6)		0(0.0)	.000
Diabetes		9(14.8)		11(18.0)	.404
ED Triage priority					.000
-Red (%)		5(8.2)		1(1.6)	
-Orange (%)		18(29.5)		7(11.5)	
-Yellow (%)		32(52.5)		25(41.0)	
-Green (%)		6(9.8)		28(45.9)	
-Blue (%)		0(0.0)		0(0.0)	
In-hospital mortality		20(32.8)		2(3.3)	.000

*DGC* decreased general condition; *ED* emergency department; *IQR* interquartil range

^a^of patients with documentation of parameter

^b^A history suggestive of a new infection and fulfilment of two or more of the following criteria: temperature < 36°C or > 38.3°C, heart rate >90 beats/min, respiratory rate >20 breaths/min, acutely altered mental status, plasma glucose >6.6 mmol/ L (unless diabetic) is considered positive for sepsis. All necessary parameters are documented

Sixty-five patients were randomly selected to the DGC reference group. Four of these were excluded for various reasons, leaving a total of 61 patients in this group (see Fig. 1). For characteristics, see Table 2.

### Time to antibiotics

**Fig. 2:**
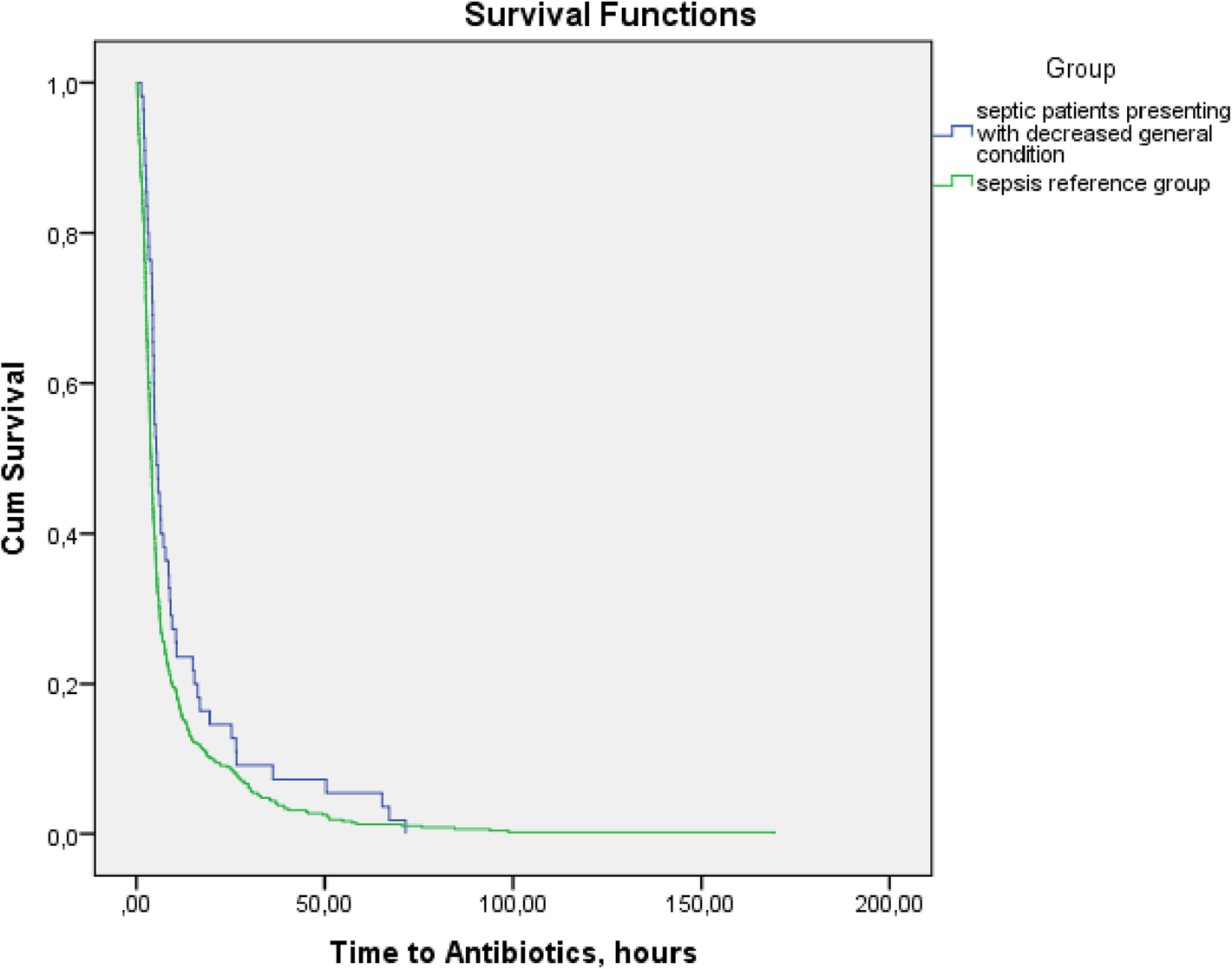
Kaplan-Meier survival curve for cumulative incidence proportion for initiation of antibiotics by group. ED = Emergency Department

The median time to antibiotics for septic patients presenting with ED chief complaint DGC was 5:26 (hours:minutes), IQR 4:00–10:40. The corresponding time for the sepsis reference group was 03:56 (hours:minutes), IQR 2:21–7:32 (*p*-value 0.001) (see Fig. 2).

### Mortality

The in-hospital mortality rate among septic patients presenting with ED chief complaint DGC was 32.8 % (20 of 61 patients). In the sepsis reference group, the corresponding rate was 10.9 %, (56 of 516 patients). The crude OR (Odds Ratio) for in-hospital mortality among septic patients presenting with ED chief complaint DGC versus that for the sepsis reference group was 4.01; 95 % CI, 2.19–7.32), see Table 3. This association remained significant when adjusted for sex, age, priority, comorbidity and fulfilment of sepsis criteria according to the Robson screening tool (OR 4.31; 95 % CI, 2.12–8.77), see Table 3. However, an interaction was found between sex and group, where the highest odds for mortality was identified among septic men presenting with ED chief complaint DGC. The model demonstrated good fit using the Hosmer-Lemeshow goodness-of-fit test (*P* = 0.38).

**Table 3:** Mortality by group and covariates among ED-patients with a discharge ICD-code consistent with sepsis. OR for in-hospital mortality by group and covariates among 577 ED-patients at Södersjukhuset with a discharge ICD-code consistent with sepsis

Variable	Category	Crude		Univariable, unadjusted		Multivariable, adjusted			
						Adjusted for all factors		Adjusted for all factors and interaction between group and sex	
		n	% dead	OR	95 % CI	OR	95 % CI	OR	95 % CI
Group	Sepsis + DGC = group 1	61	32.8	4.01	2.19–7.32	4.31	2.12–8.77	-	
	Sepsis reference group = group 2	516	10.9	Ref		Ref			
Sex	Women	239	14.6	1.24	0.77–2.02	1.16	0.65–2.07	-	
	Men	338	12.1	Ref		Ref			
Age	>75	274	18.6	3.33	1.68–6.58	5.70	2.26–14.35	6.04	2.37–15.41
	65–74	132	10.6	1.73	0.76–3.94	2.30	0.77–6.81	2.29	0.76–6.87
	<65	171	6.4	Ref		Ref		Ref	
Priority^a^	<15 min	314	16.2	1.85	1.11–3.07	2.32	1.18–4.56	2.48	1.24–4.96
	>15 min	263	9.5	Ref		Ref		Ref	
Charlson comorbidity index	≥5	78	16.7	1.48	0.68–3.24	0.89	0.33–2.40	0.82	0.29–2.27
	3–4	119	16.8	1.50	0.75–3.01	1.16	0.51–2.65	1.18	0.51–2.71
	1–2	237	11.0	0.91	0.48–1.75	0.69	0.32–1.49	0.68	0.31–1.50
	0	143	11.9	Ref		Ref		Ref	
Fulfilment of Robson ^b^	no	123	17.1	1.36	0.77–2.41	1.71	0.88–3.32	1.76	0.89–3.48
	yes	320	13.1	Ref		Ref		Ref	
Interactions group and sex	Group 1, man	39	41.0					5.23	2.18–12.52
	Group 1, woman	22	18.2					1.06	0.27–4.10
	Group 2, man	299	8.4					0.57	0.30–1.10
	Group 2, woman	217	14.3					Ref	

*ED* Emergency Department, *ICD* International Classification of Diseases, *OR* Odds Ratio, *DGC* Decreased General Condition, *CI* Confidence Interval, *Ref* Reference, *min* minutes

^a^< 15 min = Red/Orange, >15 min = Yellow/Green/Blue

^b^A history suggestive of a new infection and fulfilment of two or more of the following criteria: temperature < 36 °C or > 38.3 °C, heart rate >90 beats/min, respiratory rate >20 breaths/min, acutely altered mental status, plasma glucose >6.6 mmol/ L (unless diabetic) is considered positive for sepsis. All necessary parameters are documented

### Accuracy of the Robson screening tool and clinical judgment

#### The Robson screening tool

Thirty-four of the 54 septic patients presenting with ED chief complaint DGC having documentation for all the required Robson parameters, were considered septic in accordance with the tool, corresponding to a sensitivity of 63.0 % (95 % CI: 48.7–75.7). For the DGC reference group, 13 of 41 patients with documentation for all the required Robson parameters were considered septic in accordance to the tool, corresponding to a specificity of 68.3 % (95 % CI: 51.9–81.9) for the tool, see Table 4. Furthermore, PPV (Positive Predictive Value) for the Robson screening tool was 72.3 % (95 % CI: 57.4–84.4), NPV (Negative Predictive Value) was 58.3 % (95 % CI: 43.2–72.4), the Positive Likelihood Ratio (+LR) was 1.99 (95 % CI: 1.21–3.25) and the –LR (Negative Likelihood Ratio) was 0.54 (95 % CI: 0.36–0.81).

**Table 4:** Sensitivity and specificity of the Robson screening tool and ED doctor clinical judgment. Sensitivity and specificity of the Robson screening tool and ED doctor clinical judgment with respect to sepsis identification*, among adult ED patients presenting with DGC

	Septic patients (according to discharge ICD-code) presenting to the ED with chief complaint DGC	DGC reference group-total	DGC reference group-patients with infectious discharge diagnosis are excluded
	*n* = 61	*n* = 61	*n* = 38
The Robson screening tool**			
Sepsis according to tool	34 (true positive, **63.0 %** ^**a**^)	13 (false positive, 31.7 %)	5 (false positive, 19.2 %)
No sepsis according to tool	20 (false negative, 37.0 %)	28 (true negative, **68.3 %** ^**b**^)	21 (true negative, **80.8 %** ^**b**^)
Clinical judgment			
Sepsis according to clinical judgment	15 (true positive, **24.6 %** ^**a**^)	0 (false positive, 0.0 %)	0 (false positive, 0.0 %)
No sepsis according to clinical judgment	46 (false negative, 75.4 %)	61 (true negative, **100.0 %** ^**b**^)	38 (true negative, **100.0 %** ^**b**^)

*ED* Emergency Department, *DGC* Decreased General Condition

*Reference sepsis is defined as discharge ICD-code sepsis upon discharge and exclusion of all patients developing signs of infection ≥48 h after ED admittance

**Only patients with documentation for all required parameters for the Robson screening tool are included in the analysis

Bold characters indicate the sensitivity^a^ and specificity^b^ of the Robson screening tool and clinical judgment

If patients with infection-related discharge ICD-codes were excluded from the DGC reference group, the specificity of the tool increased to 80.8 % (95 % CI: 60.6–93.4), see Table 4.

#### Clinical judgment of sepsis by ED doctor

Fifteen of the 61 patients defined as septic in accordance to ICD-code, and presenting with ED chief complaint DGC were identified as septic by ED doctor clinical judgment, corresponding to a sensitivity of 24.6 % for clinical judgment (95 % CI: 14.5–37.3). In the DGC reference group no patients were considered septic, corresponding to a specificity of 100 % for ED doctor clinical judgment (95 % CI: 94.1–100.0), see Table 4. The PPV for clinical judgment was 100.0 % (95 % CI: 78.0–100.0), the NPV was 57.0 % (95 % CI: 47.1–66.5) and the -LR was 0.75 (95 % CI: 0.65–0.87).

## Discussion

This is, to the best of our knowledge, the first study comparing the outcome of septic patients according to ED presentation as described by chief complaint. The results show that septic patients presenting with ED chief complaint DGC have a longer time to antibiotics and an increased odds to die during hospital care, as compared to septic patients with other ED chief complaints. Second, a screening tool was compared with ED doctor clinical judgment, with respect to sepsis identification among septic patients presenting with ED chief complaint DGC. The sensitivity of the screening tool was superior, but the specificity inferior, to that of ED doctor clinical judgment.

### Time to antibiotics

Septic patients presenting with ED chief complaint DGC had a longer time to antibiotics compared to septic patients with other ED chief complaints. There are several possible reasons for this. First, is our belief that the diagnostic procedure to a higher extent is dependent on laboratory results if the clinical presentation is non-specific, which may further increase time to treatment. Second, a tendency towards lower ED triage priority was seen among septic patients presenting with ED chief complaint DGC, which may, in turn, affect time to antibiotics even if the receiving doctor suspects sepsis. However, the difference in priority was not statistically significant in the current study.

### Mortality

We found a four-fold increased odds of in-hospital mortality among septic patients presenting with ED chief complaint DGC, compared with septic patients with other ED chief complaints. There are several possible contributing factors to this. First, time to antibiotics differed between the groups, and timely antibiotic treatment has previously been shown to improve outcome for septic patients [9]. Second, less deviation of vital parameters was seen among septic patients presenting with ED chief complaint DGC. Normal vital parameters are associated with a lower level of monitoring during hospital care, which may in turn increase the risk of unnoticed deterioration. However, it has been previously described that severe sepsis can occur without deranged vital parameters [10]. Hence, an optimal level of monitoring should be considered for all septic patients.

### The Robson screening tool

The sensitivity of the Robson screening tool was superior to clinical judgment, but the specificity inferior. A higher sensitivity for the screening tool is in accordance with our previous results in the pre-hospital setting [17]. However, the sensitivity of the tool was lower in the current study (63.0 vs. 75.0 %) [17], which may be explained by the different study populations; in the current study the screening tool was applied solely to the patients presenting with ED chief complaint DGC, showing less deviation of heart rate and temperature, both included in the Robson screening tool.

Singer et al. recently evaluated an ED sepsis screening tool [22], including vital parameters and bedside lactate, the latter traditionally considered a sign of severe sepsis. A sensitivity of 34 % and a specificity of 82 % was reported for this tool. Fulfilment of the infection criteria was based on ED nurse clinical judgment which may, in addition to the lactate criteria, have contributed to a lower sensitivity for that tool.

However, Goerlich et al. reported a sensitivity of 85.7 % and a specificity of 78.4 % for an ED sepsis screening tool based on heart rate, respiratory rate, temperature and a spot check StO2 device [23]. Sepsis was defined as ED fulfilment of SIRS (Systemic Inflammatory Response Syndrome) criteria along with a source of infection, which differed from the current study where ICD-code sepsis was used. This, in addition to the fact that we assessed patients presenting to the ED with chief complaint DGC, may explain the difference in accuracy.

The high proportion of patients in the DGC reference group suffering from infections and sepsis, but not discharged with ICD-codes consistent with sepsis, could decrease the specificity of the tool in our study. It was apparent on manual chart review that patients in the DCG reference group sometimes fulfilled sepsis criteria. However, we adjusted for this by performing a separate sub analysis excluding patients with discharge ICD-codes consistent with infection from the DGC reference group. This led to an increased specificity of the tool (see Table 4).

### Clinical judgment of sepsis by ED doctor

The sensitivity of ED doctor clinical judgment in the current study exceeded that of EMS providers in our previous study [17] (24.6 vs. 11.9 %). The latter could reflect a better knowledge of sepsis presentation among ED doctors but also an increased access to laboratory results. However, the results indicate that only one fourth of the septic patients presenting to the ED with the chief complaint DGC were identified as septic by ED doctors, according to chart review. Septic patients presenting with ED chief complaint DGC had less deviation of temperature compared with the sepsis reference group, and sepsis identification has previously been shown to be associated with high temperature upon ED arrival [24]. Furthermore, when collecting data, it was often apparent in the notes that the ED doctor suspected sepsis more frequently than was explicitly expressed, e.g., the ED doctors could order antibiotics recommended for severe sepsis without literally stating the suspicion thereof. Hence, lack of documentation of suspected sepsis in ED records may have contributed to the low sensitivity of clinical judgment in the current study. The specificity of ED doctor clinical judgment was 100 % which may reflect that the ED doctor clinical judgment strongly affects what diagnosis the patient receives upon discharge from hospital.

### The study groups

Some differences were identified between the study groups. The frequency of alcohol overconsumption was significantly higher among septic patients presenting with ED chief complaint DGC compared with the sepsis reference group (see Table 1). This may indicate a decreased host response to sepsis following alcohol abuse.

Altered mental status was twice as common among septic patients presenting with ED chief complaint DGC as compared with the sepsis reference group. This may reflect that altered mental status can be the only sign of incipient sepsis and is also a criterion for severe sepsis, hence important to identify.

Surprisingly, the comorbidity score between the above mentioned groups did not differ.

## Limitations and future research

There are several limitations to this study.

First, the retrospective study design is associated with missing data.

Furthermore, defining clinical judgment by ED doctor as documentation of suspected sepsis may lead to an underestimate of the true rate of suspected sepsis, hence an overestimate of the performance of the screening tool. This problem may be solved using a prospective study design.

Moreover, the high proportion of patients with infection-related conditions within the DGC reference group contributed to a lower specificity of the Robson screening tool. However, we adjusted for this problem by excluding all patients with discharge diagnosis associated to infection in a sub analysis, and the results demonstrated an increased specificity.

In addition, the sepsis reference group consisted of all patients discharged with an ICD-code consistent with sepsis, but with chief complaints other than DGC upon ED arrival. Hence, within the sepsis reference group it is possible that other non-specific presentations are included, e.g., dizziness. However, we do not expect this to have exaggerated the main results of our study, but rather would have reduced the observed differences of time to treatment and mortality between the septic reference group and the septic group presenting with DGC.

The study sample was included on the basis of discharge ICD-code, a method used also in other studies [1, 25]. ICD codes have been shown to underestimate the number of septic patients [26]. However, the current study aims to compare time to antibiotics and mortality between two patient groups representing different presentations of sepsis. In this context the comparison between the groups is important and the absolute inclusion of all potential septic patients is expected to be less so.

Furthermore, the allocation to a specific chief complaint according to triage categories could possibly have been arbitrary in some cases. However, this reflects the real life in the ED setting where there is limited time and interpersonal experience may vary.

The Robson screening tool was created for use in the pre-hospital rather than the ED setting. However, the initial ED care is similar to the pre-hospital situation as clinical assessment is often based on bedside diagnostics.

The confidence interval for in-hospital mortality was wide, indicating a low precision of the point estimate and that the sample size was small. However, the patient sample reflects that of a large ED over one year.

Finally, this is a single centre study in an urban setting which may limit the generalizability of our results. However, the study hospital is one of the largest EDs of northern Europe, admitting patients both from the city centre of Stockholm as well as from rural areas. Hence, the results are most likely generalizable to other ED populations.

### A future screening tool

Rather than advocating that the Robson screening tool is the ultimate tool, we have interpreted the results so that they support the use of a screening tool. Conversely, the ultimate screening tool remains to be designed. An appropriate ED screening tool for sepsis could be incorporated into the current ED triage system and thus serve as support for clinical decision making. Increased ED identification of sepsis may lead not only to shorter times to antibiotic treatment, but may also enable fluid resuscitation. We believe that these factors together, could improve outcome for septic patients presenting with non-specific ED chief complaints such as DGC.

## Conclusions

Septic patients presenting with ED chief complaint DGC had a less favourable outcome, measured as longer time to antibiotics and higher in-hospital mortality, as compared with septic patients with other ED chief complaints. Our results indicate that implementation of a screening tool may increase the identification of sepsis among patients with non-specific presentations, enabling timely treatment of these patients.

## Abbreviations

+LR: Positive Likelihood Ratio
ADAPT: Adaptive Process Triage
DGC: decreased general condition
ED: Emergency Department
HCAI: Health Care Associated Infection
ICD: International Classification of Diseases
IQR: Interquartile Range
–LR: Negative Likelihood Ratio
NPV: Negative Predictive Value
OR: Odds Ratio
PPV: Positive Predictive Value
SIRS: Systemic Inflammatory Response Syndrome
SPSS: Statistical Package for the Social Sciences
